# Circumstances leading to intimate partner violence against women married as children: a qualitative study in Urban Slums of Lahore, Pakistan

**DOI:** 10.1186/s12914-015-0060-0

**Published:** 2015-08-25

**Authors:** Muazzam Nasrullah, Rubeena Zakar, Muhammad Zakria Zakar, Safdar Abbas, Rabia Safdar

**Affiliations:** Department of Public Health Medicine, School of Public Health, Bielefeld University, Bielefeld, Germany; Institute of Social and Cultural Studies, University of the Punjab, Lahore, Pakistan

## Abstract

**Background:**

Child marriage (<18 years) is prevalent in Pakistan which is associated with negative health outcomes including intimate partner violence (IPV). Our aim is to describe the types and circumstances of IPV against women who were married as children in urban slums of Lahore, Pakistan.

**Methods:**

Women of reproductive age (15–49 years) who were married prior to 18 years, for at least 5 years were recruited from most populous slum areas of Lahore, Pakistan. Themes for the interview guide were developed using published literature and everyday observations of the researchers. Interviews were conducted by trained interviewers in Urdu language and were translated into English. The interviews were tape-recorded, transcribed, analyzed and categorized into themes.

**Results:**

All 19 participants were married between 11 and 17 years. Most respondents were uneducated, poor and were working as housemaids. Majority of participants experienced verbal abuse, and threatened, attempted and completed physical violence by their husbands. A sizeable number of women reported unwanted sexual encounters by their husbands. Family affairs particularly issues with in-laws, poor house management, lack of proper care of children, bringing insufficient dowry, financial problems, an act against the will of husband, and inability to give birth to a male child were some of the reasons narrated by the participants which led to IPV against women.

**Conclusions:**

Women married as children are vulnerable to IPV. Concerted efforts are needed from all sectors of society including academia, public health experts, policy makers and civil society to end the child marriage practice in Pakistan.

## Background

Intimate Partner Violence (IPV) is a serious public health concern, worldwide [1]. IPV from male intimate partners resulted in profound negative health consequences among women [1]. Women with IPV have been reported to suffer from injuries, gynecological problems such as sexually-transmitted diseases, and psychological diseases like depression, and post-traumatic stress disorder [2]. Globally, almost 30 % of all women who have been in a relationship have experienced physical and/or sexual violence by their intimate partner [3].

IPV is prevalent in Pakistan [4–7], where around 40 % of ever-married women of reproductive age have been reported to suffer from some form of violence [8]. Of those who suffer violence, around 32 % have suffered lifetime physical violence, and 19 % experienced physical violence in the past12 months [8]. Around 11 % women suffered physical violence while they were pregnant [8]. Husbands were perpetrator in 79 % of violent cases with majority of victims being socially disadvantaged i.e. uneducated and residing in rural areas of Pakistan [8].

Child marriage practice (marriage prior to 18 years of age) is prevalent in Pakistan, which disproportionately affects young girls in rural, low income and low education households [9–11]. Child marriage practice has shown to be associated with negative health outcomes of women and children in Pakistan [9, 12] and in other low-and middle-income countries [13–17]. Further, women who are married as children have reported to suffer more violence from their husbands and in-laws as compared to those married as adults [13–15, 17–19]. The act of IPV against young and adolescent women who are married as children is prevalent in Pakistan with every third women aged 15–24 years experienced some sort IPV during their lifetime [11]. Limited knowledge is available about different types of IPV (psychological, threatened physical, attempted physical, completed physical, unwanted sex) experienced by women married as children. There is dearth of literature on circumstances that leads to IPV against women married as children. The aim of this study is therefore, to describe the types and circumstances of IPV against women who were married as children in urban slums of Lahore, Pakistan.

## Methods

### Selection of participants

The present study is a part of a broader qualitative study titled “Child marriage and its impact on maternal and child health in Pakistan”. Participants were selected for interview if the woman 1) was married before the age of 18 years (child marriage) 2) was married for at least 5 years. Reasons for setting the said selection criteria were to make sure that the interviewee had a prenatal and postnatal experience and that they have spent a considerable time in marital union. Overall in the study we had themes on “Reasons and attitude towards child marriage”, “Fertility outcomes”, “Pre- and post-natal care”, “Morbidity and mortality of children under 5 years of age” and “spousal violence”. This paper is limited to women’s experience of different types of spousal violence.

Participants were selected from urban slums of Lahore city, Pakistan. Lahore is the second most populous city in Pakistan with a population of over ten million [20]. According to the Population Association of Pakistan, 23.5 % of the population of Lahore city is comprised of females of reproductive age, and 61 % of women aged ≥ 15 years are married [21]. Multistage sampling technique was used to select the localities. During the first stage, we randomly selected two of nine administrative towns i.e., Nishtar Town and Daata Gunj Buksh Town. During the second stage, urban slums were selected randomly from the list of slums provided by Town Municipal Administration. The list by Town Municipal Administration had eight slums in Nishtar town and 7 slums in Daata Gunj Buksh Town. Of these slum areas, three areas from each town were randomly selected. Eligible participants were selected from these six slum areas with the help of gatekeepers. Gatekeepers were female representatives of non-government organizations (NGOs) that work for women’s empowerment and help women suffering with domestic violence in the locality. The NGOs representatives had a rapport and efficient networking with women in the locality and knew the cases of women who were married as children. The gatekeepers were key for recruitment of participants because of cultural barriers, and helped us in identification and recruitment of participants. The NGO workers arranged the interview time and place as per convenience of the participants because of security and confidentiality concerns. The NGO representatives identified 23 women for in-depth interviews, nevertheless, we achieved saturation point at 19th interview and stopped further interviews at that stage.

The interviews were conducted during a period of 16 weeks (June to September 2013). The majority of interviews (52.6 %; 10 of 19) were conducted at the participants’ homes when they were alone during the daytime. The remaining 9 interviews (47.4 %) were conducted at the NGO workers’ office because of privacy and convenience of the participants.

### Data collection

Themes for the interview were developed using published scientific literature and everyday observations of the researchers. For instance, the themes of “psychological violence” and “unwanted sex” were derived from available literature [6] whereas the themes “threatened physical violence” and “attempted and completed physical violence” were derived from the discussion of participants and day to day observations of the researchers.

The in-depth interview guide was drafted and revised a few times by the researchers, and later field tested on two participants. The guide was further revised based on the feedback received from these two participants. Two female interviewers, holding Masters degree in sociology were deployed for data collection. Both interviewers (one [RS] is also co-author) had more than three years of experience in conducting demographic surveys, in-depth interviews, and focus group discussions. One of the senior researchers (RZ) provided one day training to the interviewers to explain the study objectives, in-depth interview guide, and ethical considerations.

In-depth interview were deemed appropriate for obtaining rigorous opinions and detailed description of participants’ experience of IPV. The methods have been used in prior research on IPV against women [22].

The interviews were open-ended and carried out in a conversational style. In-depth semi-structured face-to-face individual interviews were conducted in a separate room away from other people to maintain the privacy of the participants. The interviewers established a rapport with the participants for 5 to 10 min before starting each session by sharing common day to day observations. Each interview was followed by a 10 min closing casual conversation. Field notes and casual encounters with participants were also noted by the interviewers during the interview. In this paper we presented our results on themes of different types of IPV (psychological, threatened physical, attempted physical, completed physical, unwanted sex), and circumstances leading to these types of IPV against women in Pakistan.

Respondent’s safety, privacy and anonymity was maintained during the recruitment and interviews of the participants, consistent with the World Health Organization guidelines [23]. After explaining the objectives of the study and rapport building, written informed consent was taken. The interviews with the participants were voluntary and they were allowed to discontinue the interview at any time. During the interview, no personal information like addresses and phone numbers were collected. Pseudonyms of the participants were used to maintain their anonymity. For maintaining the record of the participants, a unique code was assigned to each participant. The same unique code was then used on respective interview guide and audio recording. After completing and integrating the verbatim translation from audio recordings and field notes, the audio recordings were deleted to fully ensure the anonymity of participants. Whenever assistance was needed, participants were offered an information list of local formal and informal social services.

### Data management and analysis

In-depth interviews were conducted in Urdu language, as most of the participants were illiterate and were not well-versed with English. The data analysis was completed in stages. During the first stage, two researchers listened the recorded interviews and transcribed them in Urdu. Urdu transcripts were then translated into English and then back translated for accuracy and quality of translation. During translation, the colloquial, pauses, fluency and quotes of the participants were also transcribed. At second stage, the transcribed interviews, memoranda, and field notes were entered into Microsoft Excel. A coding system was organized and themes were manually interpreted. A scheme of numbers and letters were used to designate major categories and subcategories. At this stage, hard copies of coded and categorized data marked with colored pens, were shared with the interviewers and researchers for identification and removal of discrepancies. At the last stage, the transcripts were analyzed and discussed in the light of inferred themes.

The study methodology was reviewed and approved by Institutional Review Board (IRB) of University of the Punjab, Lahore, Pakistan (reference number: D/688/FBSS).

## Results

### Participant characteristics

The age range of the participants was 21 to 34 years old. All of them were married between the ages 11 and 17 years. Almost all participants (*n* = 18; 94.7 %) were married at the time of interview and were living with their husbands or in joint/extended families with their in-laws. Only 5 % (*n* = 1) of participants were separated and living with their daughters at the time of interview. The majority of the respondents (*n* = 11; 57.9 %) were migrants to the urban city from rural areas and the rest of eight (42.1 %) respondents belonged to the urban setting. Based on the occupational categories of the respondents and their husbands, fifteen respondents (78.4 %) were from low socio-economic class. Four of the respondents were from middle socio-economic class and were either housewives or working as school teachers. The majority of the respondents (*n* = 13; 68 %) were uneducated. About 15.8 % (*n* = 3) respondents had up to primary education, and the remaining 15.8 % (*n* = 3) had secondary education. More than half of the respondents (57.9 %) were working as housemaids and the remaining (*n* = 8; 42.1 %) were housewives.

### Psychological violence

A majority of women (13 of 19) narrated that their husbands humiliated and insulted them in front of others by abusing them verbal and yelling on them. Women stated that they had no alternative to get rid of that situation. Yelling occurred due to family affairs particularly issues with in-laws, instigation of mother in law, poor house management, and bringing insufficient dowry.

Once I did not invite my mother in law for lunch. She instigated her son (my husband) against me that I am careless and do not respect her. He (my husband) became violent on this issue and humiliated me in front of all family members. [Participant in early thirties, uneducated, and married at the age of 16 years]

Few women (2 of 13) stated infertility as a reason of humiliation by their husbands in front of others. It was believed by the victims that men adopt humiliating behavior to show their supremacy over women.

A sizeable number of the participants (7 of 19) reported that their husbands restricted information and movement of their wives and they did not allow them to leave house without their permission. It was further highlighted that husbands do not discuss financial matters and matters related to work with their wives.

My husband does not discuss money matters with me and he even does not allow me to ask anything about business. He says you do not need to interfere in the business or the work outside home. [Participant in early thirties having 5 years of schooling , and married at the age of 16 years]

Our data revealed that couples having the same age group or lesser difference in the age of husband and wife did not show restriction to access to information and mobility of woman. However, those with greater age differences with husband being many fold older than wife tried restricting their women at homes.

One woman who was in her late twenties and housewife said *“Men considered women as their property which has no will and could not move.”*

In contrast, a majority of women (12 of 19) reported that their husbands did not restrict information or their movement unnecessarily.

### Threatened physical violence

Almost all participants (17 of 19) reported experiencing threatened physical violence from their husbands. According to the participants, threatened physical violence was a technique by their husbands to lessen their stress at work, hide their faults, and to make wives feel inferior.

My husband used to threaten me of violence because I was young and immature. He always had bossy attitude to cover his faults. [Participant in mid-twenties, educated till 10th grade, and married at the age of 16 years]

“Children’s future” i.e. the children will have no home if spouse decided to separate and “family honor” were the two reasons narrated by the participants that forced them to maintain the spousal relationship and bear their violent husbands.

There were only two participants who reported that they never experienced threatened physical violence by their husbands. Both the couples had developed mutual trust between their husbands. Upon further probe, it was explained by one of the participant that she had never crossed her “limits” therefore, her husband never threatened her with physical violence. It was interesting to explore that “wife’s limits” were defined by her husband. One such woman who was uneducated and married at the age of 12 with 35 years old man said,

I have never crossed my limits nor did I take any decision without my husband’s permission. He is a sober person and we both respect each other. Therefore, threatening has never been an option for any issue. [Participant in thirties, uneducated, and married at the age of 12 years]

Upon asking the circumstances leading to threatened physical violence by husbands, the majority of woman (13 of 19) stated that quarrel on domestic issues, financial problems, child care problems, and anything happening against the will of husband led to their threatening behavior. Not dealing with in-laws “well” (9 of 19) and difference of opinion with husband (6 of 19) was also narrated as few of the reasons for threatening behavior of husbands. However, women (3 of 19) who did not express their difference of opinion with husbands were able to avoid violence.

### Attempted and completed physical violence

A majority of the participants (16 of 19) stated that their husband had attempted violence against them and tried to hit, slap, push and hurt them.

He has a habit of being impatient and short tempered. He has attempted hitting me and the kids many times especially at the time when he comes back home in the evening and children make a noise. [Participant in mid-forties, uneducated, and married at the age of 15 years]

In some cases (7 of 19), husbands not only attempted violence but physically hit them.

My husband not only attempted violence against me but most often he succeeded. He was a drinker [addict] and when he was drunk, he hit me. [Participant in early twenties, having 2 years of schooling, and married at the age of 13 years]

It was reported by 4 of 19 participants that some men consider themselves honored, glorious and masculine if they beat their wives. They brag their act of violence and feel proud of it.

My husband is such a strange person who thinks that beating a woman will give him honor and glory. He makes me feel inferior because of my inability of not having male child [son]. What can I do in this regard? It’s Allah’s will to grant a son or a daughter. [Participant in mid-twenties, with 10 years of schooling, and married at the age of 17 years]

Only a few women (3 of 19) told that they had not faced violence in their life possibly due to their submissive behavior with their husbands and in-laws.

Upon asking the circumstances leading to attempted and completed physical violence by husbands, the participants narrated that inability to give birth to a male child, lack of proper care of children, and arguments with in-laws led to the violent behavior of their husbands.

Once my 1 year old daughter [referring to the eldest one] fell down while playing and she was hurt. My husband was annoyed and said that why didn’t you take care of her and because of your carelessness she was severely injured. He pushed me so hard that I fell down on the table and injured my head. [Participant in early thirties, uneducated, and married at the age of 14 years]

### Unwanted sex

A sizeable number of women (8 of 19) reported unwanted sex by their partners. Intoxication, anger and sexual arousal of husbands were named some of the reasons for unwanted sex. Most unwanted sexual encounters resulted after quarrel or dispute.

Unwanted sex is also a symbol of love. It is a way to resolve the dispute between husband and wife. After having physical proximity, we discuss our disputes amicably and resolve them. [Participant in mid-twenties, had primary education, and married at the age of 17 years]

However, there were few cases (2 of 8) of unwanted sex in which women did not want sex in order to sustain pregnancy but men forcefully had sex with her. One woman who was married at the age of 14, uneducated and wife of a delivery boy stated,

I did not want sex with my husband during the early days of my pregnancy [to save my pregnancy] because I already had a miscarriage but he insisted to do it. [Participant in mid-twenties uneducated, and married at the age of 14 years]

## Discussion

Our study showed that IPV was common among women married as children. Child marriage is prevalent in south Asian countries including Pakistan [17]. The data from a nationally representative study showed that about a half of married women in Pakistan got married before the age of 18 years [8]. A brief comparison of other countries in South Asia also reveals a similar trend. In Nepal, 40 % of girls are married by the age of 15, whereas 7 % before the age of 10 years. The similar situation exists in India where median age at first marriage is 16.4 years [17].

Our participants belonged to low socio-economic class with no formal schooling. While we cannot generalize the findings but previous studies have shown that mostly women married as children poor, uneducated and reside in rural areas [17, 24]. Findings of our study also revealed that almost a half of the participants were employed in low-paid and unskilled jobs. Despite being self-employed, these women were not empowered to achieve social and economic autonomy [25]. This state of affair transcends into their marital life where they continue living in poverty [26].

Consistent with previous national and international research, our study found that IPV including sexual, physical and psychological violence is common among women married as children [25, 27–30]. Early marriage put women to insidious forms of psychological violence and they face emotional pressure by their husband and in-laws [24]. Likewise, the present study found that a majority of women were anguished when their husbands humiliated and insulted them in front of others. Psychological violence is normalized in women’s marital life as a routine matter [31]. Women married as children are at a formative stage of psychological development, and this type of IPV can result in devastating mental health outcomes for these women [32].

Due to a poor socio-economic background, a majority of participants were living in extended families that may increase their vulnerability to IPV. Women living in extended families reported many reasons of psychological violence including instigation of husband by mother-in-law, poor household management, and bringing insufficient dowry [33]. Almost similar causes of psychological violence have been highlighted in other South Asian countries [34, 35]. As a general trend, participants belonging to poor socio-economic background associate psychological violence with the lack of financial resources, and in some cases husbands’ drug addiction [36]. However, women from relatively middle social class associate psychological violence with disrespectful behavior of women with in-laws [36].

Few participants stated infertility as a reason of mental distress from their husbands. It was shown in earlier study that women married as children have limited knowledge of their responsibilities as a wife, and almost no information on sex and child birth [17]. In this context, women married at an early age remain under pressure to demonstrate fertility and in case they were unable to conceive, face a constant mental pressure that impacts these women in multiple ways [13]. Arguably, lower socio-economic status coupled with patriarchal mindset blame the woman to be infertile even when a male is at fault [37].

Findings of the present study also revealed that husbands with greater age difference with their wives exhibit controlling behaviors towards their wives. It has been reported that women married as children often had older spouses with less freedom of movement, less autonomy and decision making in reproductive and household decisions which consequently increase their vulnerability to IPV [11, 13, 24].

Our study revealed that a majority of respondents reported experiencing attempted and/or completed physical violence by hitting, slapping, pushing, or hurting. Studies conducted in diverse setting invariably indicate that women married as children are less capable than those who marry as adults, which can place them at a higher risk of experiencing physical violence [35]. Women in our study reported that, husbands felt masculine, prestigious, and honored by committing IPV against their wives. It was found that male chauvinist thinking led husbands to commit violence. However, it is argued that male chauvinism is a learned behavior that originates from home in backdrop of a traditional mindset and certain values that are inculcated in men since the early days of their life [38].

In our study a few women considered that complete obedience of husband is a way to save women from violence [25]. Such beliefs potentially undermine the concept of marital equality and egalitarian gender relations and perpetuate the acceptability of husbands’ supremacy and ultimately allow controlling behaviors and violence against women by their husbands [39]. Additionally, acceptability of patriarchal views by women increase the tolerance against violence and forces them to stay in an abusive relationship [40].

Participants of the present study highlighted several reasons which led to physical violence including inability to give birth to a male child and disgrace to family honor. Contrary to these findings, literature suggests that the most dominant causes of physical violence among women married as children in Pakistan include their lower socio-economic status, poverty, and family conflicts [13, 34].

In the present study more than a half of women reported experiencing unwanted sex by their husbands. Women married as children are more exposed to sexual violence [11]. Study from India also revealed that women married as children were more likely to expose to unwanted sex than those who married as adults [19]. These women have little say in reproductive decision making which exposed them to unwanted pregnancies and sexually transmitted infections including long-lasting effects on their mental health [24, 41].

To the best of researchers’ knowledge, this is the first qualitative study highlighting the circumstances of IPV against women who were married as children in Pakistan. This study has some limitations. Firstly, we cannot generalize these findings to a general population, and to rural and upper social class women’s violence experiences. Secondly, findings of this study were based on self-reports and we could not check its validity. Thirdly, the use of gatekeepers may have introduced a selection bias in our study.

## Conclusion

Child marriage itself is a manifestation of violence against women and violation of human rights. Our study adds to the growing literature that women married as children are vulnerable to IPV. Family affairs particularly issues with in-laws, poor house management, lack of proper care of children, bringing insufficient dowry, financial problems, an act against the will of husband, and inability to give birth to a male child were some of the reasons narrated by the participants which led to IPV against women. Low socio-economic status coupled with prevailing patriarchal norms push these women to not only bear IPV but also force them to stay in abusive marital relations.

Child marriage compromises the efforts to advance education, combat poverty, improve health indicators, and to reduce IPV. Concerted efforts and coordinated actions are needed from all sectors of society including academia, public health experts, and policy makers to end this practice in Pakistan. There is a need to raise community awareness related to negative health consequences of early marriage on women’ health, to increase investment on girls’ education, and actively involve men in violence prevention interventions.
